# Rationale and design of a multicenter prospective cohort study for the eVALuation and monitoring of HPV infections and relATEd cervical diseases in high-risk women (VALHIDATE study)

**DOI:** 10.1186/1471-2407-12-204

**Published:** 2012-05-30

**Authors:** Giovanna Orlando, Elisabetta Tanzi, Liliane Chatenoud, Maria Gramegna, Giuliano Rizzardini

**Affiliations:** 1 STD Unit, Infectious Diseases II, L Sacco University Hospital Via GB Grassi, 74 - 20157, Milan, Italy; 2 Department of Biomedical Sciences for Health, University of Milan, Milan, Italy; 3 Lifestyle Habits and Prevention Unit, Laboratory of Epidemiology, Department of Epidemiology, Pharmacological Research Institute “Mario Negri”, Milan, Italy; 4 Unità Organizzativa Governo della prevenzione e tutela sanitaria, Direzione Generale Sanità, Regione Lombardia, Milan, Italy; 5 Department of Infectious Diseases, L Sacco University Hospital, Milan, Italy

**Keywords:** Cervical cancer, Prevention, HPV, Epidemiology, Cytology screening, Molecular screening

## Abstract

**Background:**

Pap screening, an effective method for cervical cancer prevention, is now supported by molecular human papillomavirus (HPV) testing. Recently commercialised preventive vaccines also provide new tools for the primary prevention of cervical cancer. To determine appropriate prevention strategies, the Health General Direction, Lombardy Region, funded a project that aims to characterize and monitor HPV infections and related cervical diseases in high-risk women.

**Methods/design:**

VALHIDATE is a 5-year multicentre open prospective cohort study. It will recruit 7000 consenting women aged 13–65 years to provide information about the local biomolecular epidemiology of HPV infection and cervical diseases in high-risk women recruited from nine clinical centres and one faith-based organisation. The study will estimate the overall and type-specific prevalence of HPV infection and cervical abnormalities. It also aims to compare standard Pap screening with biomolecular screening, and to assist in the design of targeted regional prevention programs directed specifically at high-risk groups. Three groups of high-risk women: 1000 HIV-infected women (aged 26–65 years), 1000 recent migrant women (aged 26–65 years) and 3000 young women (aged 13–26 years) and 1 control group: 2000 women (aged 26–45 years) attending a spontaneous screening program, will be recruited. Sample sizes will be revised after the first year. Adult participants will undergo conventional cervical cytology, HPV DNA screening and genotyping. Paediatric participants will undergo HPV DNA testing and genotyping of urine samples. HPV DNA, cytological abnormalities and HPV types will be analysed according to demographic, epidemiological, behavioural, and clinical data collected in an electronic case report form. Overall and stratified prevalences will be estimated to analyse the associations between HPV infection and selected characteristics. Logistic regression models will be used to estimate crude and adjusted odds ratios. Cox proportional hazard models will be used to estimate hazard ratios over time and between groups.

**Discussion/main expected results:**

This study will provide substantial insight into HPV infections and related cervical diseases in high-risk groups and will help determine appropriate regional cervical cancer prevention strategies.

## Background

Cervical cancer screening with cervical cytology is a proven and effective secondary prevention strategy that can achieve a significant reduction in cervical cancer incidence in the industrialized world [1-3]. However, its efficacy is linked to the extent to which the target population is reached. Therefore, cervical cancer continues to be a major problem in the low- and medium-resourced countries of sub-Saharan Africa, South and South East Asia and Latin America [2,4-7]. Up to 70% of the estimated annual global burden of new cases and cervical cancer deaths occur in these countries because of their lack of an effective screening program [8]. Recently developed secondary prevention strategies show promise for scaling up prevention services in both developing countries and the industrialized world. These strategies are based on human papillomavirus (HPV) testing for high-risk HPV types, which are a necessary intermediate step for the development of cervical cancer and its precursors. The sole use of HPV DNA testing as a primary screening method has proven to be effective in randomized clinical trials conducted in industrialized countries as well as in simulation models [9-14]. Because the HPV DNA test also has a higher sensitivity and negative predictive value for grade II or higher cervical intraepithelial neoplasia (CIN) in immunocompetent women than conventional or liquid-based cytology, the extension of screening intervals to once every 3–5 years can be considered safe [15-17]. HPV testing is actually the recommended follow-up of abnormal cytology in women older than 30 years and after CIN treatment [18-20].

Recently commercialized cervical cancer vaccines, moreover, provide new tools for the primary prevention of cervical cancer with a very high rate of protection for more than eight years when used in women previously uninfected by the genotypes included in the vaccines [21-30]. The association of HPV-based screening and vaccination in the prevention of cervical cancer has been proven in randomized clinical trials, but several questions remain about the cost-effectiveness of the vaccine after sexual debut, the impact of non-vaccine HPV types and the best implementation practices [29,30]. The Italian Government (Ministry of Health) initiated a free vaccination program for 12-year-old adolescent girls on January 1, 2008. All Italian regions started the vaccination program during 2008 with the aim of reaching 95% vaccination coverage of the target group in 5 years (defined in “Intesa Stato-Regioni”, December 20th, 2007). In some regions, the free vaccine was also offered to other age groups. In Lombardy, the regional vaccination program began in September 2008 and offers free HPV vaccination to all 12-year olds girls. An extension to other age groups will be considered after an evaluation of the local burden and type of HPV infection among several risk groups and an analysis of the best overall strategy for the control of cervical cancer.

To determine appropriate strategies for cervical cancer prevention and to optimize the allocation of resources, the Health General Direction, Lombardy Region, funded a project that aims to characterize and monitor HPV infections and related cervical diseases in high-risk women across a wide range of ages.

## Methods/design

VALHIDATE (eVALuation and monitoring of HPV Infections and cervical relATEd Diseases in high-risk women) is a multicentre open cross-sectional and prospective cohort study conducted over a 5-year period (Dec. 2010–Dec. 2015) in the Lombardy Region. It aims to provide information about the local epidemiology of HPV infections and related cervical diseases as well as the distribution of HPV genotypes across ages and in high-risk groups of women.

The study was commissioned and funded by the Health General Direction of the Lombardy Region and has been approved by the ethical committees of the hospitals involved in the research. Approval for the storage of residual biological samples for further analysis was also obtained from the hospitals’ ethical committees.

The study will recruit 7000 consenting women aged 13–65 years through nine clinical centres in four general hospitals (4 gynaecology units, 3 infectious disease units, 2 paediatric units) and one faith-based voluntary organization that offers primary health care for migrants lacking health insurance.

One university virology laboratory, four microbiology laboratories and four pathology laboratories located in the hospitals will analyse the samples collected from the enrolled women.

The cross-sectional study was designed to estimate the prevalence of HPV infection, cervical abnormalities and HPV type-specific prevalence across a wide range of age and risk groups. The prospective cohort study was designed to compare the standard screening procedure (Pap test) with biomolecular HPV-testing across different age and risk groups and to assist in the design of targeted regional prevention programs directed specifically at high-risk groups.

The secondary objectives of the study are to: a) identify the geographical distributions of HPV 16 and HPV 18 variants and their association with clinical aspects; b) identify the prevalence, distribution and incidence of potential HPV 16 and HPV 18 vaccine escape mutants; c) monitor the HPV type distribution over time for an early identification of the type replacement phenomenon following the beginning of the vaccination campaign for 12-year-old girls in the Lombardy Region (September 2008).

### Informed consent

Written informed consent for participation in the study and for the conservation of the collected samples in a bio-bank will be asked from all eligible women and will be essential for participation in the study.

### Study population

#### *Control arm*

Two thousand women aged 26–45 years will be recruited from those attending a spontaneous Pap screening program (SPSW).

#### *Testing arms*

One thousand HIV-infected women (HIVW) will be recruited from those followed up for HIV infection in three infectious disease units. One thousand recent migrant women (less than 1 year) (RMW) will be recruited mostly through a faith-based organization, and 3000 young women (aged 13–26 years) will be recruited from those asking for a Gynaecologic (1326G) or Paediatric consultation (1318P). Those vaccinated with anti-HPV vaccines prior to enrolment or during the follow-up period will not be excluded from the study. Instead, the vaccinated participants will be considered a subgroup to be monitored and evaluated over time.

#### *Exclusion criteria*

Those with a history of histologically proven grade II or higher CIN requiring treatment, who were pregnant at the time of enrolment or who were unable to provide informed consent are excluded from the study. For participants aged < 18 years, written informed consent will be required from their parents or legal guardian.

#### *Recruitment and follow-up periods*

Participants will be recruited for 12 months; this will be extended if necessary. They will be followed up for 3 years, and the study is scheduled for completion 3 years after the last enrolment.

### Baseline evaluation and follow-up

Adult participants will undergo a medical assessment appropriate to their group (gynaecologic evaluation, and infectious disease evaluation for HIV-infected participants), cytological screening following the Italian Guidelines [31] for Cervical Cancer or algorithms modified for HIV-infected women, and HPV DNA testing. Colposcopy referral will be driven by cytology results. The conventional Pap smears will be evaluated according to the 2001 Bethesda System by four pathology laboratories.

HPV-DNA testing will be performed by the reference virology laboratory. HPV DNA-positive samples will be genotyped using a commercial kit in the four microbiology laboratories. Oncogenic risk associated with HPV types will be defined according to the 2011 IARC classification [32]. Samples with a positive HPV DNA test but with genotypes not included in the InnoLipa panel will be typed by the restriction fragments length polymorphism (RFLP) technique in the reference virology laboratory. In this reference laboratory, HPV 16- and HPV 18-positive cervical brushes or urine samples will be further subjected to a phylogenetic analysis to study the circulation of variants belonging to different geographical clusters (European and non-European).

An additional analysis for the characterization of HPV 16 and HPV 18 variants that could potentially elude the immune response induced by the vaccination (escape mutants) will be performed.

Participants with discordant results (HPV-positive/cytology-negative) will be recalled after 12 months for reassessment with cytology and HPV testing.

**Figure 1:**
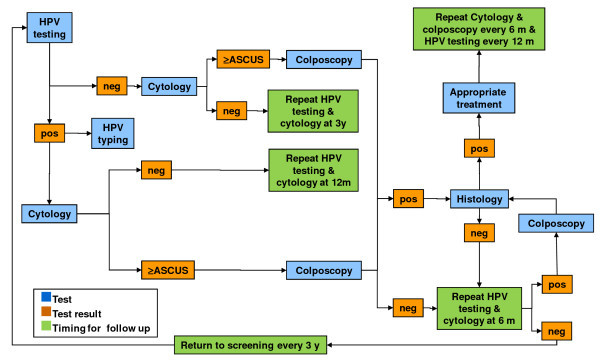
**Algorithm for the screening and follow-up of SPSW and RMW.** SPSW: women aged 26–65 years attending a gynaecologic visit for spontaneous screening. RMW: recently migrated women aged 26–65 years asking for medical assessment of any health problem. HPV testing: Nucleic acid extraction from cervical samples (by NucliSENS*®* EasyMAG™, bioMérieux bv, France) followed by PCR amplification of a 450-base pair fragment of ORF L1 using the degenerate primer pair ELSI-f and ELSI-r.

**Figure 2:**
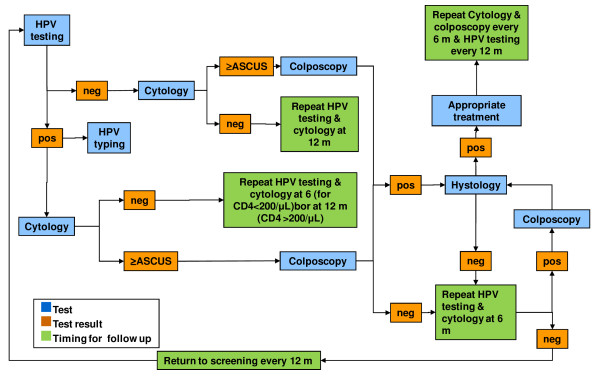
**Algorithm for the screening and follow-up of HIVW.** HIVW: HIV-infected women aged 26–65 years. HPV testing: Nucleic acid extraction from cervical samples (by NucliSENS*®* EasyMAG™, bioMérieux bv, France) followed by PCR amplification of a 450-base pair fragment of ORF L1 using the degenerate primer pair ELSI-f and ELSI-r.

**Figure 3:**
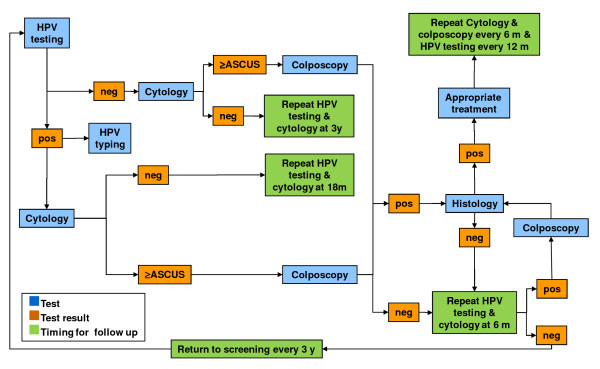
**Algorithm for the screening and follow-up of 1326G.** 1326G: young women aged 13–26 years asking for a gynaecologic assessment. HPV testing: Nucleic acid extraction from cervical samples (by NucliSENS*®* EasyMAG™, bioMérieux bv, France) followed by PCR amplification of a 450-base pair fragment of ORF L1 using the degenerate primer pair ELSI-f and ELSI-r.

**Figure 4:**
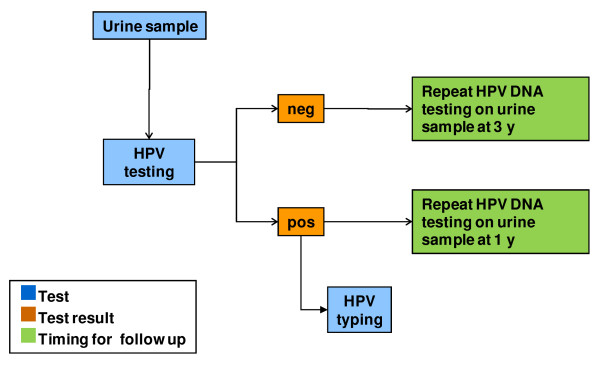
**Algorithm for the screening and follow-up of 1318P.** 1318P: young girls aged 13–18 years asking for a paediatric assessment of any health problem. HPV testing: Nucleic acid extraction from cervical samples (by NucliSENS*®* EasyMAG™, bioMérieux bv, France) followed by PCR amplification of a 450-base pair fragment of ORF L1 using the degenerate primer pair ELSI-f and ELSI-r.

Paediatric participants will undergo a paediatric evaluation and HPV DNA testing of urine samples. The algorithms for the follow-up of adult and paediatric participants are presented in Figure 1, Figure 2, Figure 3, and Figure 4. All data collected during the visits and derived from cytology and biomolecular evaluations will be recorded on a specially developed secure electronic case report form (eCRF).

### Conventional pap smears

The Pap smear sample will be obtained by using a brush (Cytobrush Plus Medscand® Medical AB, Sweden). Cervical specimens for conventional cytology will be processed by spreading the cells onto a glass slide, fixing cells within a few minutes, and then sending them to the reference pathology laboratory for investigation.

### Sample collection for molecular tests

The same cervical brush used for cytology will be then immersed and rinsed in a vial filled with 20 ml of PreservCyt® solution (ThinPrep Pap Test, Hologic Italia Srl), and stored at room temperature (RT) until processing. Ten millilitres of each PreservCyt solution containing cervical cells will be centrifuged at 3800 × g for 15 min at RT. After centrifugation, the pellet will be resuspended in 1 ml of phosphate buffered saline (PBS), transferred into a new 1.5 ml collection test tube and stored at −20 °C until nucleic acid isolation.

First-void urine samples will be collected in sterile containers, kept at RT and processed within 6–8 hours of collection. At least 15 ml of each urine sample will be centrifuged at 3800 × g for 20 min at RT. After centrifugation, the opaque phase will be recovered, transferred into a new 1.5 ml collection test tube and then centrifuged at 16,000 × g for 15 min at RT. The pellets obtained will be resuspended in 700 μl of PBS and stored at −20 °C until nucleic acid isolation.

### Colposcopy

Referral for colposcopy will be driven by the Pap results. Cytology showing atypical squamous cells or worse will be referred for colposcopy evaluation and biopsies will be taken from suspicious areas of the cervix, according to the standard procedures in Italy [33]. Biopsy results will be reported as normal, CIN grade I, II, III, or as invasive cancer, according to the international criteria [34].

### Nucleic acid extraction and HPV DNA detection

DNA will be extracted from cervical and urine samples using an automated system for total nucleic acid extraction (NucliSENS*®* EasyMAG™, bioMérieux bv, France) according to the manufacturer’s instructions. The concentration and purity of the extracted DNA will be evaluated through a Thermo Scientific NanoDrop 2000 spectrophotometer (Thermo Fisher Scientific Inc., France). DNA integrity will be assessed by amplification of a 268-base pair (bp) fragment in the ubiquitous β-globin gene using the primer pair PCO_4_ (5′–CAACTTCATCCACgTTCACC–3′) and GH_20_ (5′–gAAgAgCCAAggACAggTAC–3c′) [35].

HPV DNA will be detected by PCR amplification of a 450-bp segment of ORF L1 using the degenerate primer pair ELSI-f (5′–gCNCARggHCATAAYAATgg–3′) and ELSI-r (5′–CgNCCHAADggAAAYTgATC–3′)[36]. An equimolar mixture of each primer will be added to the PCR master mix to a final concentration of 30 pmol. PCR will be performed using GoTaq® DNA Polymerase (Promega, Madison WI, USA) according to the manufacturer’s instructions. The ELSI-f and ELSI-r primers will be used with the following amplification reaction conditions: 5 min denaturation at 94 °C followed by 40 cycles of amplification. Each cycle will consist of a denaturation step at 94 °C for 30 sec, an annealing step at 55 °C for 30 sec and an elongation step at 72 °C for 30 sec. The last cycle will be followed by a 7 min elongation step at 72 °C. Each PCR run will include positive controls (DNA extracted from HPV 16-positive cells, Caski) and negative (water) control samples. The amplification products will be revealed using an electrophoresis analysis of 2% agarose gels containing ethidium bromide. Amplified product bands will be compared with molecular weight standards (DNA Molecular Weight, Marker 100, Sigma, St. Louis, MO).

### HPV genotyping

The 450-bp fragments will be genotyped using INNO-LiPA HPV Genotyping *Extra* (Innogenetics N.V., Belgium), a line probe assay based on the principle of reverse hybridization. Part of the L1 region of the HPV genome will be amplified using SPF10 primers. The resulting biotinylated amplicons will be subjected to denaturation and hybridization with sequence-specific DNA probes for the identification of 28 different HPV genotypes: 6, 11, 16, 18, 26, 31, 33, 35, 39, 40, 43, 44, 45, 51, 52, 53, 54, 56, 58, 59, 66, 68, 69, 70, 71, 73, 74, and 82. To establish the genotype/s present in the sample, the line patterns will be compared with the interpretation chart supplied with the assay.

### HPV X

Samples for which the obtained line pattern cannot be assigned to any genotype pattern or that have no type-specific lines, but at least one positive HPV control line will be scored as HPV-positive, but untypeable (HPV X). These samples will be referred to the reference virology laboratory for genotype analysis by RFLP. The 450-bp fragments obtained by the ELSI-PCR protocol will be tested by the RFLP technique with RsaI, DdeI and HaeIII digestion enzymes (New England Biolabs, Ipswich, MA). Briefly, 10 μl of the amplified products will be added to three final mixtures of 15 μl containing 1U of each enzyme in a specific buffer. The restriction reaction will be performed at 37 °C for 1 hour and the enzymes will be then inactivated at 80 °C for 20 minutes. Fragments of different lengths will be separated on 3% agarose gel. The pattern of fragments generated by the restriction enzymes will allow for identification of all types in the genus *alpha* of Papillomaviruses.

### Phylogenetic analysis of HPV 16 and HPV 18 LCR variants

For all HPV16- and HPV18-positive samples, a 730-bp fragment of the LCR region will be amplified (HPV 16 primer sets: 16-LCRs 5′–gCTTgTgTAACTATTgTgTCA–3′ and 16-LCR-as 5′–gCTTgTgTAACTATTgTgTCA–3′; HPV 18 primer sets: 18-LCRs 5′–ACCTgCCAAgCgTgTgCgTg–3′ and 18-LCR-as 5′–AggTgCCTgCggTgCCAgA–3′) [37,38]. The LCR multiple sequence alignment will be conducted using ClustalX [39]. Sequence editing will be carried out by means of BioEdit software [40] to compare the nucleotide sequences with those of reference strains obtained from the on-line database. Phylogenetic trees will be constructed using the MEGA package [41], and a bootstrap analysis [42] will be performed. HPV 16 variants will be classified into the four major phylogenetic branches: European (East Asia), Asian-American, African-1 and African-2, as described by Yamada et al. [43,44]. HPV 18 variants will be classified into two major phylogenetic branches: European (East Asia) and non-European (Asian-American, African-1, African-2).

### HPV 16 and HPV 18 escape mutants

For all HPV 16-positive samples, a 911-bp and 879-bp fragment of the L1 gene will be amplified (1° fragment: L1_16_1F 5′–ATgTCTCTTTggCTgCCTAg–3′ and L1_16_1R 5′–gCATCAgAggTAACCATAgAAC–3′; 2° fragment: L1_16_2F 5’-CTATggACTTTACTACATTACAggCTA-3’; L1_16_2R 5′–gTTTAgCAgTTgTAgAggTAgATgA–3′) [45]. The overlapping of the sequences obtained from the two amplified fragments will allow the analysis of the L1 gene (1498-bp).

For all HPV 18-positive samples, a 911-bp and 870-bp fragment of the L1 gene will be amplified (1° fragment: L1_18_1F 5′–ATggCTWTgTggCggCCTAg–3′ and L1_18_1R 5′–gAgTCAgAggTAACAATAgAgC–3′; 2° fragment: L1_18_2F 5′–CCAYggRCTTTAgTACATTgCAAgATA–3′ and L1_18_2R 5′–gTTTAgAAgACgTAgYggCAgATgg–3′) [45]. The overlapping of the sequences will allow the analysis of the L1 gene (1489-bp).

Phylogenetic analysis of the sequences will be conducted by the bioinformatics programs described above. The analysis of codon-specific non-synonymous and synonymous substitutions and the selection pressures will be inferred by MEGA [41] and the Datamonkey facility [46].

### Bio-bank

Residual biological samples collected in the PreservCyt solution that are positive for HPV DNA and/or for cytological abnormalities will be stored at −20 °C for further studies. Concordant HPV DNA-negative/cytology-negative samples and residual urine samples will be processed as follows: after centrifugation, the pellet obtained will be stored at −20 °C and will then be available for future molecular investigations

### Variables to be analysed and data security

Descriptive statistics (means ± SD or median) and frequency analyses will be used to describe the whole study sample and each cohort with regard to demographic, epidemiological, behavioural, clinical, immunological, and HPV vaccination status data. Data will be collected with a specially developed e-CRF released on a web platform built with PHP 5.3 and JQuery. The database resides on MySql 5.0. The application is hosted at the Mario Negri Institute (99% uptime guaranteed, according to the service level agreement) with security features according to the current laws. Anonymous data extraction will be performed at the data storage centre.

### Statistical analyses

#### *Cross-sectional study*

Overall and HPV type-specific prevalence will be estimated with 95% confidence intervals (CI), according to the Poisson approximation, in the four participant groups (three high-risk groups and control group). Logistic regression models will be used to estimates odds ratios (OR) and their corresponding 95% CI to quantify the associations between HPV infection and major demographic, clinical and behavioural characteristics in the four participant groups. All variables will be analysed in both univariate and multivariate models to control for major confounding factors.

Sensitivity, specificity, and positive and negative predictive values of the HPV test compared with the Pap test will be estimated overall and by group. Test performance in matched pairs will be estimated with McNemar’s tests, and proportions will be compared using Fisher’s exact tests. Exact methods will be applied when expected cell frequencies are less than five.

#### *Power calculation*

The sample size was calculated by taking published data as well as economic feasibility into account to allow the estimation of differences in the prevalence of type-specific HPV between the high-risk and control groups. From preliminary results obtained from the first 1300 women enrolled (717 SPSW, 290 HIVW, 160 RMW and 133 1326 G), the prevalence varied from 0.5%–2% (for major high-risk HPV genotypes: 16, 18, 31, 51, 52, 53) in the control group, and was 2–3 fold higher in the high-risk groups (for HPV 16, 31, 51, 52, 53). The planed sample of 1000 women for each high-risk group and 2000 low-risk women will be adequate to detect differences for genotypes with a prevalence of 1%–1.5% in the control group.

#### *Prospective study*

We will analyze the incidence of new cytological abnormalities, progression or regression of baseline lesions, incidence, clearance and persistence of HPV infection for all women who had at least one follow-up visit. The proportion of women lost to follow-up is expected to be different in each participant group. The RMW group may have more difficulty in returning for a follow-up visit. Socio-demographic differences between women who return and who are lost to follow-up will be analysed. Conversely, women in the HIV cohort, who will be recruited from the centre they attend for HIV treatment, may be more likely to attend the study follow-up visits.

The proportions of women who we expect to be lost to follow-up are therefore estimated at around 10%, 20% and 60% in the HIVW, SPSW and RMW groups, respectively.

Single and multiple infections will be defined without considering uncharacterized HPV types (HPV X). The Kaplan-Meier method will be used to evaluate differences in the incidence of abnormal cytology at follow-up between women in the high-risk groups versus the control group. When appropriate, a Cox model will be used to account for the effects of major confounding factors. For the paediatric group, we will recruit about 250 girls for each year of age between 13 and 18 years to estimate the age at first HPV infection, time to clearance or persistence of HPV infection, and the change after 3 years of follow-up related to major behavioural characteristics.

All the analyses will be performed using SAS software for Windows version 9.2 (SAS Institute Inc., Cary).

## Discussion/Main expected results and impact

In the last 50 years, the introduction of screening methods has substantially reduced the incidence of cervical cancer in the industrialised world. However, inequalities in the distribution of cervical cancer prevalence and mortality are still present at national, regional or local levels. In general, high-risk women are those who are not reached for any reason by prevention and screening programs. This study will provide substantial insight on HPV infections and related cervical diseases, which will enable us to define the risk of cervical cancer and its precursor lesions in high-risk women compared with the general population living in the Lombardy Region. In turn, this information will assist health care providers and policy makers to improve regional strategies and optimize cervical cancer prevention in high-risk groups.

## Abbreviations

VALHIDATE: eVALuation and monitoring of HPV Infections and relATEd cervical diseases in high-risk women; CIN: Cervical Intraepithelial Neoplasia; SPsW: Spontaneous Pap Screening Program Women; HIVW: HIV infected women; RMW: Recent Migrant Women; 1326G: women aged 13–26 referred to gynaecologic consultation; 1326P: women aged 13–26 referred to paediatric consultation; RFLP: Restriction Fragment Length Polymorphism; eCRF: electronic Case Report Form; RT: Room Temperature; PBS: Phosphate Buffered Saline.

## Competing interests

The authors declare that they have no competing interests.

## Authors’ contributions

GO, ET, MG, GR contributed to the conception and design of the study. GO, LC, ET conceived, designed and refined the data collection process and database structure and helped in developing the eCRF. GO, GR, MG, ET secured funding for the study. LC, PB will develop and conduct the statistical analysis. AMC, CG, MF will perform cytology. NZ, GL, GC will perform HPV genotyping. ET, SB will perform molecular and phylogenetic studies. MMF, IC, GZ, EB, IA, VB, SE, GT, and AM will recruit participants and carry out the screening and follow-up processes, contributing substantially to the acquisition of clinical and epidemiological data. All authors read, revised and approved the final manuscript.

## VALHIDATE Study Group

Maria Concetta Antonacci, Pathology Unit, L Sacco University Hospital, Milan, Italy - antonacci.concetta@hsacco.it

Irene Arcidiacono, Division of Infectious and Tropical Diseases, Hospital of Lodi, Italy - irene.arcidiacono@ao.lodi.it

Paola Bertuccio, Lifestyle Habits and Prevention Unit, Laboratory of Epidemiology, Department of Epidemiology, Pharmacological Research Institute “Mario Negri”, Milan, Italy - paola.bertuccio@marionegri.it

Silvia Bianchi, Department of Biomedical Sciences for Health, University of Milan, Italy - silvia.bianchi@unimi.it

Veronica Boero, Fondazione IRCCS Ca’ Granda Ospedale Maggiore Policlinico, Mangiagalli, Regina Elena Milan, Italy - vboero@gmail.com

Giuseppe Cambiè, SIMT, Hospital of Lodi - giuseppe.cambie@ao.lodi.it

Irene Cetin, Gynaecology Unit, L Sacco University Hospital, Milan, Italy - Irene.cetin@unimi.it

Susanna Esposito, Department of Maternal and Pediatric Sciences, Università degli Studi di Milano, Fondazione IRCCS Ca’ Granda Ospedale Maggiore Policlinico, Milan, Italy - susanna.esposito@unimi.it

Marcella Falchetti, Department of Patology- Spedali Civili di Brescia, - marcella.falchetti@spedalicivili.brescia.it

Michela Maria Fasolo, STD Unit, Infectious Diseases II, L Sacco University Hospital, Milan, Italy - fasolo.michela@hsacco.it

Carlo Galli, Pathology Unit, Hospital of Lodi - carlo.galli@ao.lodi.it

Emanuela Bertazzoli, Hospital of Lodi, Italy - emanuela.bertazzoli@virgilio.it

Giovanna Lunghi, Virology Unit, Clinical Chemistry and Microbiology Lab - Ca’ Granda Hospital, Milan - lab.viro@policlinico.mi.it

Alberto Matteelli, Institute of Infectious and Tropical Diseases, University of Brescia, Italy - matteelli@med.unibs.it

Giancarlo Tisi, Gynaecology Unit, Spedali Civili Brescia, Italy - dott.tisi@gmail.com

Nadia Zanchetta, Microbiology Unit, L Sacco University Hospital, Milan, Italy - zanchetta.nadia@hsacco.it

Gianvincenzo Zuccotti, Pediatric Unit, L. Sacco Hospital, Università degli Studi di Milano, Milan, Italy - gianvincenzo.zuccotti@unimi.it

## Study protocol

Sacco Hospital Ethical Committee resolution n35 (3^rd^ Feb 2010), Sacco Hosp General Direction Resolution n174/2010 (9 March 2010).

## Pre-publication history

The pre-publication history for this paper can be accessed here:

http://www.biomedcentral.com/1471-2407/12/204/prepub
